# Exercise-induced hypoalgesia following HIIT: associations with cardiac interoceptive accuracy and the moderation by physical fitness

**DOI:** 10.3389/fpsyg.2026.1953847

**Published:** 2026-10-12

**Authors:** Maria Geisler, Marco Herbsleb, Alexander Ritter, Feliberto de la Cruz, Yubraj Gupta, Andy Schumann, Ilona Croy, Karl-Jürgen Bär

**Affiliations:** 1 Department of Psychosomatic Medicine and Psychotherapy, University Hospital Jena, Jena, Germany; 2 Department of Sports Medicine and Health Promotion, Friedrich-Schiller-University Jena, Jena, Germany; 3 Department of Clinical Psychology, Friedrich-Schiller-University Jena, Jena, Germany; 4 Department of Psychotherapy and Psychosomatic Medicine, Faculty of Medicine and University Hospital Carl Gustav Carus, TUD Dresden University of Technology, Dresden, Germany; 5 German Center for Mental Health (DZPG), Site Jena-Magdeburg, Halle, Germany

**Keywords:** aerobic fitness, athletes, exercise-induced hypoalgesia, high-intensity interval training, interoception, pain modulation, VO_2_max

## Abstract

**Introduction:**

Exercise-induced hypoalgesia (EIH) refers to the reduction in pain perception following acute exercise, yet the roles of aerobic fitness and interoception in this response remain unclear.

**Methods:**

This study investigated pain perception and cardiac interoceptive accuracy before and after high-intensity interval training (HIIT) in endurance athletes and non-athletes. Forty-one healthy adults (20 athletes, 21 non-athletes) completed a maximal cardiopulmonary exercise test to determine VO₂max and self-report measures, including the Multidimensional Assessment of Interoceptive Awareness (MAIA). On a second study day, participants underwent pressure pain testing and a HBDT before and after HIIT. Training status was operationalized both categorically (athletes vs. non-athletes) and continuously (VO₂max).

**Results:**

Athletes showed greater EIH than non-athletes, reflected in a significant reduction in pain following HIIT in athletes only and a larger pain change score compared with non-athletes. Higher VO₂max was likewise associated with greater pain reduction. Baseline cardiac interoceptive accuracy was positively associated with VO₂max, and interoceptive accuracy decreased significantly from pre- to post-exercise in both groups, with a more pronounced decline in athletes. MAIA scores showed no significant associations. VO₂max also significantly moderated the relation between post-exercise changes in cardiac interoceptive accuracy and pain reduction, suggesting that the link between interoceptive change and EIH differed as a function of aerobic fitness.

**Discussion:**

From an adaptation-based, hormesis-informed perspective, the findings are compatible with a distinction between acute cost and longer-term adaptation: intense exercise may transiently reduce cardiac interoceptive accuracy, while repeated exposure may be associated with better baseline interoceptive accuracy and more effective post-exercise pain modulation.

## 1 Introduction

Exercise-induced hypoalgesia (EIH) refers to the acute reduction in pain perception following physical exercise and is a well-established phenomenon in healthy individuals (Naugle et al., 2012; Rice et al., 2019). Experimental studies have shown that EIH can be elicited by different exercise modalities, including aerobic exercise, resistance training, and high-intensity interval training (HIIT), with more intense exercise often producing stronger hypoalgesic effects (Vaegter and Jones, 2020). Although EIH is considered a robust group-level effect, its magnitude varies substantially between individuals. Previous research suggests that training status is one factor contributing to this variability. Athletes often show stronger pain attenuation after exercise than non-athletes (Geisler et al., 2026), whereas individuals with chronic pain frequently display reduced or absent EIH (Naugle et al., 2012; Rice et al., 2019). These differences indicate that exercise-related pain modulation is shaped by individual characteristics and prior bodily experience. Several mechanisms have been proposed, including endogenous pain inhibition, opioid and endocannabinoid activity, and central pain modulation processes (Geisler et al., 2020a; Scheef et al., 2012; Vaegter and Jones, 2020). These mechanisms provide important explanations for how EIH may be generated, but substantial interindividual variability in the magnitude of EIH remains, raising the question of which individual characteristics may contribute to this variability (Rice et al., 2019).

One potentially relevant, but still understudied, factor is interoception. Interoception refers to the perception and processing of internal bodily signals such as heartbeat, breathing, and visceral sensations (Craig, 2002). It is increasingly understood as a central process for integrating bodily states with affective and regulatory functions. Because pain is itself a bodily signal with strong motivational relevance, interoceptive processes may be important for understanding individual differences in pain perception and modulation.

There are several reasons to assume a link between interoception and EIH. First, exercise strongly alters internal bodily signals by increasing cardiovascular and respiratory activity, thereby changing the interoceptive context in which pain is processed (Wallman-Jones et al., 2021). Second, individuals differ in their ability to perceive such bodily signals accurately. In particular, endurance athletes have been reported to show higher interoceptive accuracy than non-athletes, especially for cardiac signals, although findings are not entirely consistent (Kleber et al., 2025; Seabury et al., 2023; Wallman-Jones et al., 2021; Zeng et al., 2025). The relationship between exercise and interoception is bidirectional: exercise changes the intensity and salience of internal bodily signals, while interoceptive processing helps regulate effort, fatigue, pacing, and the subjective experience of exertion (McMorris et al., 2018; Wallman-Jones et al., 2021).

From a theoretical perspective, acute high-intensity exercise may reduce the precision of interoceptive processing because multiple salient bodily signals compete for limited attentional resources. In this case, pain may become less distinct or less salient within the overall bodily state, which could contribute to reduced pain perception after exercise. An alternative prediction is that higher or improved interoceptive accuracy after exercise may be associated with reduced EIH, as more precise perception of bodily signals could also maintain or enhance awareness of painful sensations rather than dampening them. Accordingly, both stable between-person differences and acute exercise-induced changes in interoception may be relevant for explaining EIH. Despite these theoretical considerations, the relationship between interoception and EIH has received little direct empirical attention. In particular, it remains unclear whether interoceptive accuracy differs between athletes and non-athletes in the context of acute exercise and whether changes in interoceptive accuracy are associated with exercise-induced changes in pain perception.

The present study investigated pain perception and cardiac interoceptive accuracy before and after a bout of HIIT in endurance athletes and non-athletes. Interoceptive accuracy was assessed using the heartbeat detection task (HBDT). Training status was operationalized both categorically (athletes vs. non-athletes) and continuously by VO_2_max, the latter to capture individual differences in aerobic fitness beyond group membership. We hypothesized that (H1) HIIT would induce EIH, with greater pain reduction in individuals with higher training status; (H2) higher training status would be associated with greater interoceptive accuracy; and (H3) exercise-induced changes in interoceptive accuracy would be associated with changes in pain perception following HIIT. We further tested whether training status moderates the association between changes in interoceptive accuracy and EIH.

## 2 Materials and methods

### 2.1 Ethical approval

Written informed consent was obtained from all participants before the study. This study conformed to the standards set by the Declaration of Helsinki, except for registration in a database, and was approved by the Ethics Committee of the Faculty of Social and Behavioral Sciences, Friedrich Schiller University Jena (FSV 23/006).

### 2.2 Participants

The present analyses were based on data from the same overarching study protocol as reported in Geisler et al. (2026), with a partially overlapping sample. Whereas the previous report focused on cortical responses assessed using functional near-infrared spectroscopy (fNIRS), the present study examined behavioral pain responses and cardiac interoceptive accuracy before and after HIIT. Participants were recruited through University of Jena advertisements, social networks dedicated to runners and triathletes, and local running and triathlon clubs in Jena and nearby areas. Inclusion criteria were aged ≥18 years, and no history of pain disorders, psychiatric, or neurological illnesses. Additionally, endurance athletes had engaged in at least 6 hours of endurance sports per week for three or more years, while non-athletes had not participate in any sports over the last year. The final sample comprised 41 healthy adults, including 20 endurance athletes and 21 non-athletes. Groups did not differ in age, with athletes aged 28.7 ± 8.5 years and non-athletes 28.7 ± 9.0 years. Athletes had a lower body mass index (BMI; 22.2 ± 2.0 kg/m^2^) than non-athletes (24.4 ± 4.1 kg/m^2^). Sex distribution was comparable between groups, with 7 women and 13 men in the athlete group and 7 women and 14 men in the non-athlete group. As expected, athletes reported substantially more weekly training (9.05 ± 3.15 h/week) than non-athletes (0.05 ± 0.22 h/week). Cardiorespiratory fitness also differed markedly between groups: relative peak oxygen uptake was 53.9 ± 9.3 mL·kg^−1^·min^−1^ in athletes and 33.9 ± 5.3 mL·kg^−1^·min^−1^ in non-athletes. Participants received €20 compensation.

### 2.3 Study design

Participants completed two laboratory sessions. On day 1, cardiorespiratory fitness was assessed by means of a cardiopulmonary exercise test (CPX) on a cycle ergometer to determine maximal oxygen uptake (VO_2_max) and peak power output (PPO). On day 2, participants completed the EIH paradigm, consisting of a pre-exercise pain assessment, a bout of HIIT, and a post-exercise pain assessment. In addition, cardiac interoceptive accuracy was assessed immediately before and after the EIH paradigm using the heartbeat detection task (HBDT; Schandry, 1981). Between sessions, participants completed a battery of online questionnaires assessing psychological and interoceptive traits. These included the State and Trait Anxiety Inventory (STAI-G; Laux et al., 1981), the Life Orientation Test-Revised (LOT-R; Glaesmer et al., 2008), the Pain Catastrophizing Scale (PCS; Meyer et al., 2008), the Multidimensional Assessment of Interoceptive Awareness (MAIA; Mehling et al., 2012), the Big Five Inventory-10 (BFI-10; Rammstedt et al., 2013), and the Patient Health Questionnaire (PHQ-9; Spitzer et al., 1999), and the Positive and Negative Affect Schedule (PANAS; Janke and Glöckner-Rist, 2012), which was administered before and after the HIIT session to assess momentary affective state, (see Supplementary Table S1, Figure 1).

**Figure 1:**
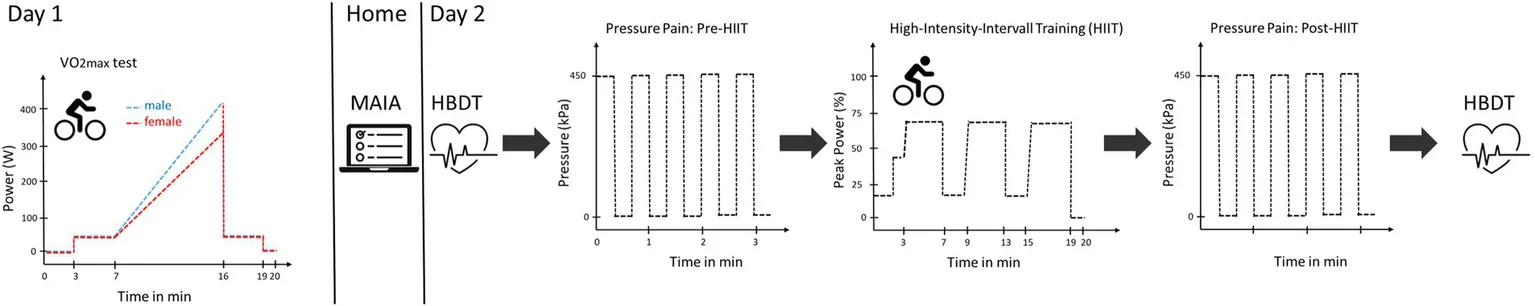
Study design. On Day 1, participants completed a VO₂max test, followed by self-report questionnaires at home, including the MAIA. On Day 2, cardiac interoceptive accuracy (HBDT) and pain sensitivity (pressure pain test) were assessed before and after a bout of HIIT. VO₂max, maximal oxygen uptake; MAIA, Multidimensional Assessment of Interoceptive Awareness; HBDT, heartbeat detection task; HIIT, high-intensity interval training.

#### 2.3.1 Day 1: cardiopulmonary exercise test (CPX)

Cardiorespiratory fitness was assessed on day 1 using a symptom-limited CPX on a cycle ergometer. Respiratory gas exchange was recorded continuously, and VO_2_max and PPO were derived from the test. PPO was subsequently used to individualize exercise intensity during the HIIT session on day 2. Further details of the CPX protocol are provided in Geisler et al. (2026).

#### 2.3.2 Day 2: exercise-induced-hypoalgesia paradigm and HIIT

The EIH paradigm was administered on day 2 and consisted of a pre-exercise pressure-pain assessment, a bout of HIIT, and a post-exercise pressure-pain assessment. Pressure pain was assessed at the thenar muscle using a pressure algometer (SENSEBox, Somedic AB, Hörby, Sweden). To familiarize participants with the procedure while minimizing habituation or sensitization, a brief non-painful demonstration trial was conducted before the experimental stimulation. Painful stimulation was then applied five times to one hand before HIIT and repeated five times on the contralateral hand after HIIT to reduce local sensitization effects. Each stimulus was applied over a 1 cm^2^ area for 20 s, with a 20 s rest period between stimuli, and participants rated the intensity of each stimulus during the rest period on a numeric rating scale ranging from 0 (“no pain”) to 100 (“unbearable pain”). Pressure intensity was set to 450 hPa in athletes and 400 hPa in non-athletes in order to achieve comparable subjective pain levels across groups, given prior evidence of lower pain sensitivity in athletes.

HIIT was performed on a cycle ergometer and individualized based on PPO derived from CPX. The protocol consisted of a 3-min warm-up (2 min at 20% PPO, 1 min at 40% PPO), followed by three 4-min high-intensity intervals at 70% PPO, each separated by 2-min active recovery periods at 20% PPO. Heart rate, gas exchange, and perceived exertion were monitored during exercise. After HIIT, participants were given a brief pause to hydrate and wipe sweat from their hands before the post-exercise pain assessment began. This assessment started approximately 2–3 min after completion HIIT.

A detailed description of the pain stimulation and HIIT procedures is available in Geisler et al. (2026).

#### 2.3.3 Heartbeat detection task

Cardiac interoceptive accuracy was assessed on day 2 using the HBDT (Schandry, 1981). The task was administered twice in a seated position: once before the pre-exercise pain assessment and once after the post-exercise pain assessment, with the latter taking place approximately 10 min after HIIT.

At each assessment, participants completed three trials with durations of 20, 30, and 40 s. Trial order was randomized, but the same order was retained across the pre- and post-exercise assessments within each participant to ensure comparability across time points. Thus, each participant completed a total of six HBDT trials across the session. Interoceptive accuracy scores for pre- and post-exercise were calculated as mean of the three corresponding trials using the following formula: $1-(\frac{\left(|\text{nbeatsreal}-\text{nbeatsreported}|\right)}{\text{nbeatsreal}})$ , (Schandry, 1981) and change in interoceptive accuracy was defined as post minus pre.

### 2.4 Statistical analyses

All statistical analyses were performed using R version 3.4.1 (Team, 2017). Change scores were calculated as post-exercise minus pre-exercise values for both pain and cardiac interoceptive accuracy. Accordingly, a negative EIH score indicated reduced pain following HIIT, whereas a positive interoceptive change score indicated an increase in interoceptive accuracy from pre- to post-exercise.

Descriptive statistics were computed separately for athletes and non-athletes. Group differences in baseline characteristics were examined using independent-samples *t* tests where assumptions of normality were met and Wilcoxon rank-sum tests where they were not. Differences in sex distribution were analyzed using chi-square tests and Fisher’s exact tests, as appropriate. Normality was assessed using Shapiro–Wilk tests.

Analyses directly corresponding to hypotheses H1–H3 were treated as primary confirmatory analyses. All additional subgroup-specific correlations, sensitivity analyses, and *post hoc*/subscale analyses were considered secondary or exploratory and are interpreted accordingly.

To test *H1*, pain ratings before and after HIIT were analyzed using a linear mixed-effects model with time (pre, post), group, and their interaction as fixed effects and participant as a random intercept. This model constituted the primary test of whether HIIT induced EIH and whether this effect differed between athletes and non-athletes. As a complementary analysis reflecting training status on a continuous dimension, a Spearman rank correlation was computed between VO₂max and pain change scores across the full sample. Follow-up paired-samples *t* tests within each group and the independent-samples *t* test on EIH change scores were conducted to further characterize significant effects. Exploratory subgroup-specific correlations between VO₂max and pain change were additionally examined separately within athletes and non-athletes.

To test *H2*, heartbeat-detection performance was analyzed using a linear mixed-effects model with time (pre, post), group, and their interaction as fixed effects and participant as a random intercept. Group differences in pre-exercise heartbeat-detection performance and associations between VO₂max and baseline heartbeat-detection performance were used as the primary tests of whether higher training status was associated with greater cardiac interoceptive accuracy. To examine whether the findings were robust to potential heart-rate-related influences on task performance, an additional model including heart rate as a time-varying covariate was estimated as a sensitivity analysis. Follow-up paired-samples *t* tests within each group and group comparisons of interoceptive change scores were conducted to further characterize pre-post effects. Self-reported interoceptive sensibility was analyzed using the MAIA total score as a secondary analysis, whereas MAIA subscale analyses and subgroup-specific VO₂max correlations were treated as exploratory.

To test *H3*, participant-level linear regression models were computed to examine whether exercise-induced changes in heartbeat-detection performance predicted EIH and whether this association was moderated by training status. First, moderation by categorical group membership was tested by entering interoceptive change, group, and their interaction as predictors of pain change scores. Second, moderation by aerobic fitness was tested in a separate model including mean-centered interoceptive change, mean-centered VO₂max, and their interaction term. For interpretability, predictors involved in interaction terms with VO₂max were mean-centered prior to analysis. In addition, zero-order correlations between interoceptive change and pain change scores across the full sample and within each group were examined as descriptive secondary analyses.

All tests were two-tailed, and statistical significance was set at *p* < 0.05.

## 3 Results

### 3.1 Demographic data

In accordance with our selection criteria and as shown in Supplementary Table S1, athletes and non-athletes differed in all measured parameters of endurance capacity (higher VO_2_max and higher PPO attained during the CPX). However, both groups exercised at comparable relative intensities, reaching nearly the same percentage of their individual VO_2_max during the final HIIT interval (athletes: 93.2% vs. non-athletes: 94.3%; *p* = 0.556).

Analysis of psychometric questionnaires revealed significant group differences in depression (PHQ9: athletes < non-athletes), and conscientiousness (BFI10: athletes > non-athletes). No significant group differences were observed for the remaining questionnaire measures, including positive and negative affect assessed with the PANAS before and after HIIT. For detailed statistics, please see Supplementary Table S1.

### 3.2 Exercise-induced-hypoalgesia (EIH)

As the primary hypothesis-driven test of H1, pain ratings before and after HIIT were analyzed using a linear mixed-effects model with time (pre, post), group, and their interaction as fixed effects and participant as a random intercept. The analysis revealed no significant main effect of group, *F*(1, 39) = 2.01, *p* = 0.165, η^2^ = 0.05, and no significant main effect of time, *F*(1, 39) = 2.58, *p* = 0.116, η^2^ = 0.06. However, the group × time interaction was significant, *F*(1, 39) = 6.90, *p* = 0.012, η^2^ = 0.15, indicating that pre-post changes in pain ratings differed between athletes and non-athletes, see Figure 2A.

**Figure 2:**
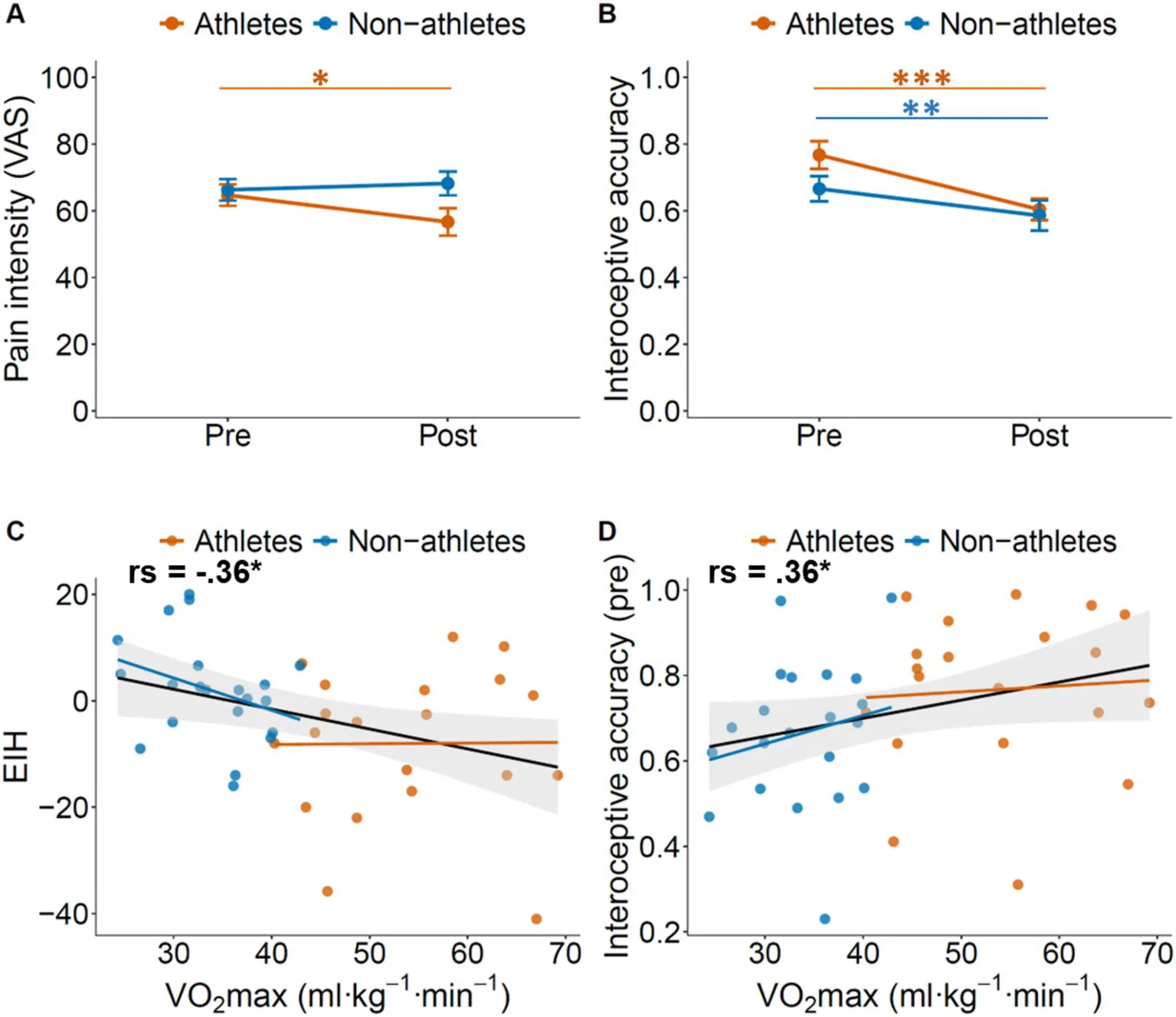
Pain and interoception in response to high-intensity interval training (HIIT) and their associations with aerobic fitness. Panels **(A)** and **(B)** show mean pain ratings and mean cardiac interoceptive accuracy (operationalized as heartbeat-detection task scores) before and after HIIT in athletes and non-athletes; error bars represent standard errors. Athletes showed a significant reduction in pain following HIIT, whereas non-athletes did not. Interoceptive accuracy decreased from pre- to post-exercise in both groups, with athletes showing overall higher interoceptive accuracy scores than non-athletes. Panels **(C)** and **(D)** show the associations of VO₂max with exercise-induced hypoalgesia (EIH), defined as pain change post-HIIT minus pre-HIIT, and with baseline interoceptive accuracy (heartbeat-detection task scores), respectively. Higher VO₂max was associated with greater pain reduction following HIIT and with better baseline interoceptive accuracy. Shaded areas indicate 95% confidence intervals. EIH = exercise-induced hypoalgesia; r_s_ = Spearman’s rank correlation coefficient. Asterisks indicate statistical significance (*p < 0.05, **p < 0.01, ***p < 0.001).

As follow-up analyses to characterize this interaction, paired-samples t tests were conducted to further characterize pre-post changes within each group. In non-athletes, pain ratings did not significantly change from pre- to post-HIIT, t(20) = 0.91, *p* = 0.372, mean difference = 1.93, 95% CI [−2.49, 6.35]. In athletes, however, pain ratings decreased significantly following HIIT, t(19) = −2.52, *p* = 0.021, mean difference = −8.03, 95% CI [−14.70, −1.36].

As an additional between-group characterization of this effect, an independent-samples *t* test was performed on the EIH change score. This analysis showed a significant between-group difference [*t*(33.31) = 2.60, *p* = 0.014, *d* = 0.90, 95% CI [0.18, 1.61]], with athletes showing a greater reduction in pain than non-athletes.

As a complementary hypothesis-relevant analysis operationalizing training status continuously, a Spearman correlation was computed between VO_2_max and pain change. Across the full sample, higher aerobic fitness was significantly associated with greater pain reduction following HIIT (r_s_ = −0.36, *p* = 0.019). Exploratory within-group analyses, however, did not reveal significant associations in either non-athletes (*r* = −0.33, *p* = 0.147) or athletes (*r* = 0.01, *p* = 0.972), see Figure 2C.

### 3.3 Interoception

To address H2, the primary analysis of post-exercise changes in heartbeat-detection performance used a linear mixed-effects model with time (pre, post), group, and their interaction as fixed effects and participant as a random intercept. The model revealed a significant main effect of time, *F*(1, 39) = 35.40, *p* < 0.001, η^2^ = 0.48, indicating heartbeat-detection performance decreased from pre- to post-exercise, see Figure 2B. The main effect of group was not significant, *F*(1, 39) = 1.33, *p* = 0.257, η^2^ = 0.03. Importantly, the group × time interaction was significant, *F*(1, 39) = 4.12, *p* = 0.049, η^2^ = 0.10, suggesting that the pre-post decrease differed between groups and was more pronounced in athletes than in non-athletes.

As a sensitivity analysis, an additional model including heart rate as a time-varying covariate was estimated to examine the potential influence of heart rate on heartbeat-detection performance. Because this more complex model did not provide a significantly better fit than the simpler model, χ^2^(1) = 2.40, *p* = 0.121, it is reported only in the Supplementary Tables S2, S3. In this adjusted model, the fixed effects of time and the group × time interaction were attenuated relative to the primary analysis.

As follow-up analyses to characterize the pre-post effect, paired-samples *t*-tests showed that heartbeat-detection performance was significantly lower after HIIT than before HIIT in both groups. In athletes, heartbeat-detection performance decreased significantly from pre- to post-exercise, *t*(19) = −4.86, *p* < 0.001, mean difference = −0.16, 95% CI [−0.23, −0.09]. Likewise, non-athletes also showed a significant decrease, *t*(20) = −3.35, *p* = 0.003, mean difference = −0.08, 95% CI [−0.13, −0.03].

As a primary hypothesis-relevant analysis of training status on a continuous dimension, associations with aerobic fitness were examined using Spearman correlations because VO_2_max deviated from normality. Higher VO_2_max was significantly associated with better baseline heartbeat-detection performance (r_s_ = 0.36, *p* = 0.019), see Figure 2D.

As a secondary analysis, self-reported interoceptive sensibility, group differences in MAIA total score were not statistically significant (*t*(38.55) = −1.75, *p* = 0.088, d = −0.56, 95% CI [−1.20, 0.08]), although athletes showed descriptively higher values than non-athletes (athletes: *M* = 24.35; non-athletes: *M* = 22.38), see Figure 3A. The correlation between VO₂max and MAIA total score was likewise positive but not statistically significant across the full sample (r_s_ = 0.28, *p* = 0.074). Exploratory within-group analyses also did not reveal significant associations in either athletes (r = 0.09, *p* = 0.700) or non-athletes (r = −0.13, *p* = 0.560), see Figure 3B. No significant group differences were observed for any MAIA subscale in exploratory *post hoc* analyses (see Figure 3C).

**Figure 3:**
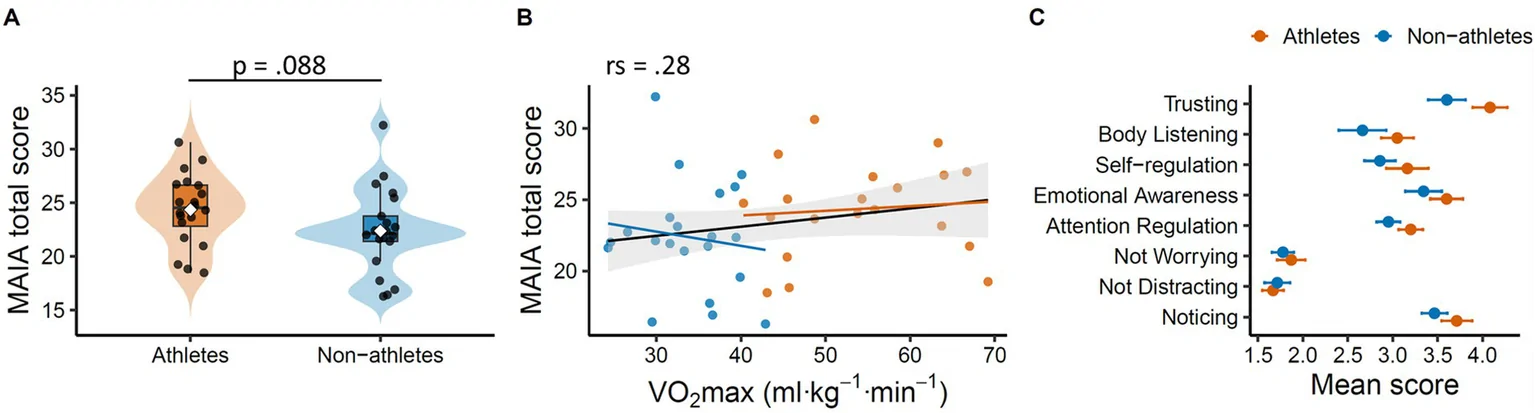
Self-reported interoceptive sensibility assessed with the Multidimensional Assessment of Interoceptive Awareness (MAIA). Panel **(A)** shows the distribution of MAIA total scores in athletes and non-athletes using violin and boxplots. Panel **(B)** shows the association between aerobic fitness (VO₂max) and MAIA total score. Panel **(C)** shows mean scores for all MAIA subscales in athletes and non-athletes; error bars represent standard errors. No significant group differences were observed for the MAIA total score or for any MAIA subscale, and the positive association between VO₂max and MAIA total score did not reach statistical significance.

### 3.4 Regression analyses

To test H3, the primary hypothesis-driven analyses consisted of participant-level linear regression models examining whether exercise-induced changes in heartbeat-detection performance predicted EIH and whether this association was moderated by training status.

As descriptive secondary analyses, zero-order correlations between change in heartbeat-detection performance and EIH were first examined. At the bivariate level, change in heartbeat-detection performance was not associated with EIH across the full sample, *r* = 0.00, *p* = 0.979, nor within non-athletes, *r* = 0.10, *p* = 0.669, or athletes, *r* = −0.25, *p* = 0.282.

In the first regression model, moderation by group membership was tested by including the interaction between change in heartbeat-detection performance and group (see Figure 4A). The overall model was significant, *F*(3, 37) = 2.89, *p* = 0.049, R^2^ = 0.19. There was a significant main effect of group, b = 14.59, SE = 5.26, *p* = 0.009, η^2^ = 0.17, indicating overall differences in EIH between athletes and non-athletes. However, neither the main effect of change in heartbeat-detection performance, b = −8.77, SE = 24.79, *p* = 0.726, η^2^ < 0.001, nor the interaction between change in heartbeat-detection performance and group, b = −32.79, SE = 30.99, *p* = 0.297, η^2^ = 0.03, was significant. Thus, group membership did not significantly moderate the association between exercise-induced changes in heartbeat-detection performance and EIH.

**Figure 4:**
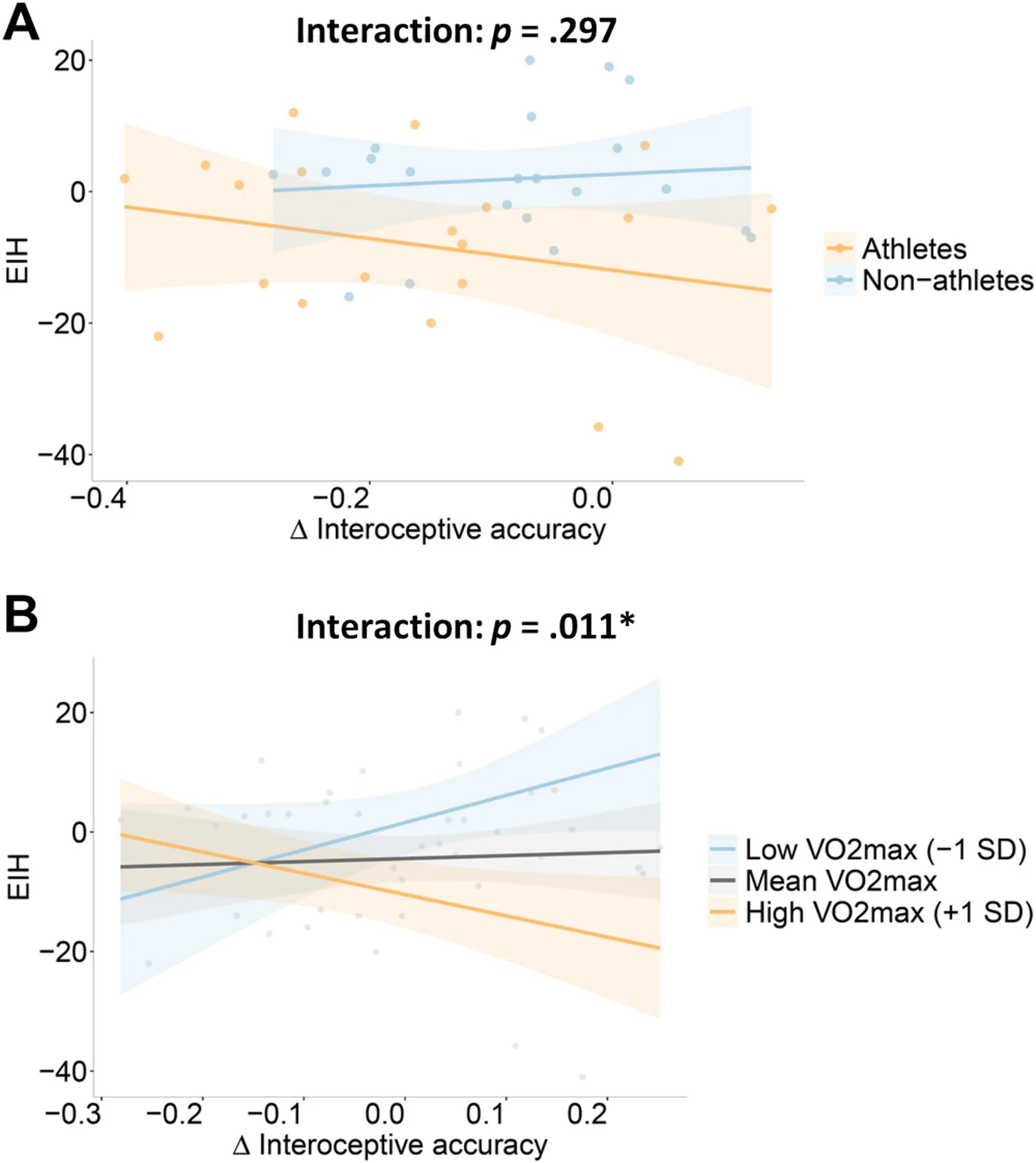
Moderation of the association between exercise-induced changes in interoceptive accuracy and exercise-induced hypoalgesia (EIH). Panel **(A)** shows the first regression model testing whether group status (athletes vs. non-athletes) moderated the association between change in interoceptive accuracy (operationalized as the pre- to post-exercise change in heartbeat-detection task performance) and EIH. Regression lines are shown separately for athletes (orange) and non-athletes (blue). The interaction was not statistically significant, indicating that the association between interoceptive change and EIH did not differ significantly between groups. Panel **(B)** shows the second regression model testing whether aerobic fitness moderated this association. Regression lines are displayed for low, mean, and high VO₂max levels (−1 SD, mean, +1 SD). Here, the interaction between change in interoceptive accuracy and VO₂max was statistically significant, indicating that aerobic fitness moderated the relation between interoceptive change and EIH. Shaded areas represent 95% confidence intervals. EIH = exercise-induced hypoalgesia; VO₂max = maximal oxygen uptake; n.s., not significant; SD, standard deviation; *p < 0.05.

In the second regression model, moderation by aerobic fitness was tested by including the interaction between mean-centered change in heartbeat-detection performance and mean-centered VO₂max (see Figure 4B). This model was significant, *F*(3, 37) = 4.81, *p* = 0.006, R^2^ = 0.28. There was no significant main effect of change in heartbeat-detection performance, b = 4.94, SE = 14.99, *p* = 0.743, η^2^ < 0.001, but a significant negative main effect of VO₂max, b = −0.48, SE = 0.15, *p* = 0.003, η^2^ = 0.16. Importantly, the interaction between change in heartbeat-detection performance and VO₂max was significant, b = −3.23, SE = 1.20, *p* = 0.011, η^2^ = 0.16, indicating that aerobic fitness moderated the association between exercise-induced change in heartbeat-detection performance and EIH. Given that EIH was operationalized as post-exercise pain minus pre-exercise pain, lower values reflected greater pain reduction following HIIT. Change in heartbeat-detection performance was likewise calculated as post-exercise minus pre-exercise values, such that lower values reflected a decline in heartbeat-detection performance after exercise. The negative interaction therefore suggests that at higher levels of aerobic fitness, greater decreases in heartbeat-detection performance were associated with smaller EIH, whereas more stable or improved heartbeat-detection performance tended to be associated with greater EIH.

## 4 Discussion and conclusions

The present study examined whether aerobic fitness and interoception help explain individual differences in exercise-induced hypoalgesia following HIIT. Overall, the findings support the view that EIH is not a uniform response to intense exercise but varies as a function of fitness-related characteristics and interoceptive processes.

First, the results suggest that aerobic fitness is relevant for the magnitude of hypoalgesic responses to exercise. Although HIIT did not produce a uniform reduction in pain across the whole sample, athletes showed clearer pain attenuation than non-athletes, and across the full sample, higher aerobic fitness was associated with greater pain reduction, although this association was not evident within either group separately. This pattern fits well with previous work describing EIH as a robust phenomenon at the group level, but one that shows substantial interindividual variability (Naugle et al., 2012; Nold et al., 2025; Rice et al., 2019). It is also consistent with reports that physically trained individuals may exhibit stronger exercise-related pain inhibition than less trained individuals (Geisler et al., 2026; Vaegter and Jones, 2020). In line with this interpretation, aerobic fitness may reflect adaptations in endogenous pain modulation or a greater capacity to tolerate and regulate intense bodily strain during exercise (Geisler et al., 2020b; Rice et al., 2019; Vaegter and Jones, 2020). More generally, this view is compatible with evidence that athletes and non-athletes can differ across multiple pain-relevant systems, including neural pain processing and autonomic network characteristics (De La Cruz et al., 2022; Geisler et al., 2021). An additional factor that may be relevant in this context is mood. Acute physical activity is known to influence affective state (Strasser and Fuchs, 2015), and mood can in turn modulate pain perception (Tang et al., 2008). Thus, affective responses to HIIT may represent another pathway contributing to variability in exercise-induced hypoalgesia. In the present study, affect was assessed with the PANAS before and after HIIT; however, we observed no significant pre–post changes or group differences in positive or negative affect (see Supplementary Table S1). Thus, our data do not suggest that affective change was a major driver of the observed group differences in EIH, even though mood remains a plausible accompanying factor in exercise-related pain modulation.

Although VO₂max was analyzed as a continuous measure, athletes and non-athletes differed markedly in aerobic fitness. Consequently, the association observed across the full sample should not be interpreted as clear evidence for a continuous dose–response relationship between aerobic fitness and EIH. Instead, it may partly reflect between-group differences between athletes and non-athletes. Exploratory within-group correlations were not statistically significant, although these analyses should be interpreted cautiously given the small group-specific sample sizes and the restricted VO₂max range within each group.

Second, the findings provide partial support for the idea that training status is linked to interoceptive functioning. Higher aerobic fitness was associated with better baseline cardiac interoceptive accuracy across the full sample, which is consistent with the assumption that repeated exposure to cardiovascular strain may sharpen perception of internal bodily signals over time (Wallman-Jones et al., 2021). At the same time, self-reported interoceptive sensibility was not clearly related to fitness. This dissociation is in line with multidimensional models of interoception and supports the distinction between objective interoceptive accuracy and subjective interoceptive sensibility (Garfinkel et al., 2015). Thus, aerobic fitness may be more specifically associated with performance-based sensitivity to bodily signals than with global self-reports of body awareness. Additionally, interoceptive functioning in athletes may not be shaped by physical training alone. Recent evidence showed that mindfulness-based training in athletes was associated with parallel increases in self-reported interoceptive awareness and heart-rate variability, suggesting a potential link between interoceptive processes and autonomic regulation beyond exercise status per se (Campo et al., 2026). Although mindfulness-related factors and HRV were not assessed in the present study, this work highlights that group differences in interoceptive functioning may also reflect broader self-regulatory or autonomic adaptations.

A particularly noteworthy finding was that performance on the HBDT declined after HIIT in both groups. This may seem counterintuitive given the assumption that exercise increases the salience of bodily signals and could therefore facilitate interoceptive processing (Wallman-Jones et al., 2021). However, the present findings are compatible with the alternative idea outlined in the introduction: under conditions of very intense exercise, multiple highly salient bodily signals may compete for limited attentional and regulatory resources. In this context, lower post-exercise heartbeat-detection performance may reflect reduced precision in processing specific cardiac cues, but it may also be influenced by concurrent physiological activation, estimation strategies, attentional shifts, or fatigue. Earlier work has likewise suggested that the effects of physical arousal on interoceptive performance may depend on exercise intensity and timing of assessment (Montgomery et al., 1984). From a broader theoretical perspective, this transient decline may also be viewed as being conceptually compatible with a hormesis-based framework. Such models propose that acute physiological stress can temporarily impair ongoing functioning while, with repeated exposure, contributing to longer-term adaptation and an expansion of the individual’s adaptive range (Peake et al., 2015; Radak et al., 2017). In this sense, HIIT may have imposed a short-term strain on heartbeat-detection performance, even if repeated exposure to intense exercise could, in principle, be associated with more efficient regulation of bodily states over time. However, this interpretation remains speculative in the context of the present study, as our design did not assess longitudinal adaptation to repeated HIIT exposure. Importantly, this framework does not imply that fitter individuals should necessarily show a smaller acute decline in post-exercise task performance. Rather, fitness-related adaptation may be reflected more in baseline functioning or in the functional significance of post-exercise changes than in the immediate direction of the acute effect itself. This interpretation may help reconcile the present pattern, in which interoceptive accuracy declined after HIIT overall and particularly strongly in athletes, while higher aerobic fitness was nevertheless associated with better baseline interoceptive accuracy across the full sample. At the same time, the observed decline should be interpreted cautiously, as cardiac interoception tasks may also be influenced by the concurrent physiological state in which they are performed (Desmedt et al., 2018; Zamariola et al., 2018). In a supplementary model including heart rate as a time-varying covariate, the main effect of time and the group × time interaction were attenuated relative to the primary analysis. This pattern suggests that post-exercise cardiovascular activation may have contributed meaningfully to the lower post-HIIT scores. At the same time, inclusion of heart rate did not significantly improve model fit, indicating that heart rate alone is unlikely to fully explain the observed change. Accordingly, the post-exercise reduction should not be interpreted as a process-pure change in cardiac interoceptive accuracy, but rather as a pattern that may partly reflect concurrent physiological activation alongside interoceptive and attentional processes. Accordingly, the meaning of post-exercise changes in cardiac interoceptive accuracy is likely not unidimensional. This may help explain why the link between interoceptive change and pain modulation was less direct than hypothesized. Acute changes in cardiac interoceptive accuracy were not directly associated with EIH across the sample, nor was this association clearly different between athletes and non-athletes. However, aerobic fitness significantly moderated the association between post-exercise changes in cardiac interoceptive accuracy and EIH. Inspection of the interaction suggests that the functional meaning of exercise-induced changes in cardiac interoception may differ as a function of aerobic fitness. Specifically, at higher levels of aerobic fitness, greater declines in interoceptive accuracy tended to be associated with smaller hypoalgesic effects, whereas more stable or improved interoceptive accuracy tended to coincide with greater pain reduction. At lower levels of aerobic fitness, this pattern was weaker and appeared to shift in the opposite direction.

One possible interpretation is that, in fitter individuals, maintaining a more stable representation of internal bodily signals after intense exercise may reflect a more regulated post-exercise state, which may in turn be more compatible with pronounced hypoalgesia. Conversely, stronger declines in cardiac interoceptive accuracy may indicate that the acute demands of HIIT disrupted bodily signal processing to a greater extent, which may be associated with less effective pain attenuation. This interpretation is broadly compatible with the idea that exercise changes the interoceptive context in which pain is processed (Craig, 2002; Wallman-Jones et al., 2021). In less fit individuals, by contrast, the functional meaning of interoceptive change may differ. For example, a decline in interoceptive accuracy may more strongly reflect fatigue, task-related difficulty, or unspecific physiological disruption, rather than processes directly related to pain modulation.

More broadly, these findings fit the bidirectional relationship between exercise and interoception described in the literature (McMorris et al., 2018; Wallman-Jones et al., 2021). Exercise alters bodily signals, while interoceptive processing shapes how these signals are interpreted and regulated. The present results suggest that pain modulation after exercise may emerge not only from exercise intensity or endogenous inhibitory mechanisms alone, but also from how the acute bodily state is processed under conditions of high physiological demand. This may help explain why established physiological accounts of EIH, including endogenous pain inhibition and opioid- or endocannabinoid-related mechanisms, do not fully capture interindividual variability in hypoalgesic responses (Vaegter and Jones, 2020).

Importantly, the present data do not allow conclusions about causal direction. It remains unclear whether acute changes in interoceptive processing influence pain modulation after exercise, whether pain-related processes shape post-exercise interoceptive performance, or whether both are jointly affected by a third factor such as physiological strain, fatigue, or regulatory capacity. The observed interaction therefore indicates conditional covariation rather than a demonstrated mechanism.

Several limitations should be considered. No formal *a priori* power analysis was conducted for the present study; rather, the sample size was determined within the framework of the overarching study protocol and informed by previous experimental work using comparable athlete–non-athlete designs. A sensitivity analysis based on the final sample of 20 endurance athletes and 21 non-athletes indicated that, for a two-sided independent-samples t test with α = 0.05, the study had approximately 80% power to detect a between-group effect of Cohen’s d = 0.90. Thus, the present sample was primarily sensitive to relatively large effects and was not sufficiently powered to reliably detect small-to-moderate effects. This limitation is particularly relevant for the moderation analyses. The cross-sectional design does not permit causal conclusions about training effects. In addition, interoception was assessed only in the cardiac domain and with a single task. This measure has well-known conceptual and methodological limitations, as performance may be influenced not only by interoceptive processing but also by beliefs about heart rate, estimation strategies, attentional demands, and concurrent physiological state (Desmedt et al., 2018; Ring and Brener, 2018; Zamariola et al., 2018). These issues are particularly relevant in the immediate post-exercise context, where fatigue and cardiovascular arousal may affect task performance independently of interoceptive ability. Beyond these measurement-related limitations, the present findings concern acute responses to a specific HIIT protocol and may not generalize to other exercise modalities or intensities.

## 5 Conclusion

In sum, the present findings suggest that aerobic fitness is related to stronger exercise-induced hypoalgesia and to differences in cardiac interoceptive performance, whereas acute HIIT may transiently reduce post-exercise interoceptive accuracy. The association between interoceptive change and pain reduction was not consistent across the sample, but appeared to depend on aerobic fitness. These findings do not support a simple one-directional link between interoception and EIH; instead, they suggest that the role of interoceptive processes in post-exercise pain modulation may depend on the broader physiological context.

## Data Availability

The raw data supporting the conclusions of this article will be made available by the authors, without undue reservation.
