# Supplementary material for: Exercise-induced hypoalgesia following HIIT: associations with cardiac interoceptive accuracy and the moderation by physical fitness
Source: Front Psychol. 2026 Oct 12;17:1953847. doi: 10.3389/fpsyg.2026.1953847 (PMC13721068; doi:10.3389/fpsyg.2026.1953847)
Supplement: Supplementary file 1 [file Table_1.docx]

Supplements

**Table S1. Demographic characteristics of subjects**

|  | **athletes** | | **non-athletes** | |  |
| --- | --- | --- | --- | --- | --- |
|  | ***M*** | ***SD*** | ***M*** | ***SD*** | ***p-value*** |
| ***biographical data***  age (years)  BMI (kg*•*m^-^²)  endurance sport (h/week)  *cardiorespiratory fitness*  $\dot{V}$O_2max_ (ml*•*kg^-1^*•*min^-1^)  PPO (W)  ***questionnaires***  depression (PHQ 9)  anxiety (STAI G X2)  interoception (MAIA sum score)  pain catastrophizing (PCS)  extraversion (BFI 10)  agreeableness (BFI 10)  conscientiousness (BFI 10)  emotional_stability (BFI 10)  openness (BFI 10)  pessimism (LOT R)  optimism (LOT R)  positive affect pre (PANAS)  positive affect post (PANAS)  negative affect pre (PANAS)  negative affect post (PANAS) | 28.7  22.2  9.1  53.9  373.9  6.1  37.3  24.3  30.4  3.6  3.3  4.1  3.1  3.5  7.3  7.3  3.3  3.5  2.0  1.9 | 8.5  2.0  3.1  9.3  91.6  6.1  7.7  3.3  8.9  1.0  0.9  0.8  0.9  1.2  2.0  2.4  0.7  0.8  0.4  0.2 | 28.7  24.4  0.0  33.9  232.5  9.7  43.0  22.4  30.2  3.4  3.5  3.4  3.4  3.4  7.7  8.0  3.1  3.2  2.0  2.0 | 9.0  4.1  0.2  5.2  53.0  2.1  10.8  3.9  10.9  1.2  0.8  0.9  1.0  1.2  1.7  2.2  0.7  0.8  0.5  0.5 | 0.990^§^  **0.036**  **<0.001^§^**  **<0.001**  **<0.001**  **0.019**  0.060  0.089  0.959  0.936^§^  0.466^§^  **0.012^§^**  0.322^§^  0.862^§^  0.055^§^  0.305  0.385  0.315  0.977  0.563^§^ |

Group-specific mean (M) and standard deviation (SD) of demographic variables in athletes and non-athletes. Group differences were assessed using independent sample t-tests or Mann-Whitney tests (§) for non-normally distributed data (all df = 40). Significant p-values (< 0.05) are highlighted in bold. BMI = Body Mass Index, $\dot{V}$O_2max_ = maximal oxygen uptake, PPO = Peak Power Output, HIIT_VO_2_ = Oxygen uptake during High-Intensity Intervall Training (measured at the end of Intervall 3), $\dot{V}$E/$\dot{V}$O₂ = Ventilatory equivalent for oxygen at the end of Intervall 3(amount of air ventilated to consume one liter of oxygen), $\dot{V}$E/$\dot{V}$CO₂ = Ventilatory equivalent for carbon dioxide at the end of Intervall 3 (amount of air ventilated to eliminate one liter of carbon dioxide), TV = Tidal volume at the end of Intervall 3 (normalized for body surface area), PHQ-9 = Patient Health Questionnaire – 9, STAI-G-X2 = State-Trait Anxiety Inventory – Trait scale, MAIA_sum = Sum score of the Multidimensional Assessment of Interoceptive Awareness, PCS = Pain Catastrophizing Scale, BFI-10 = Big-Five-Inventory -10 item version, LOT-R = Life-Orientation-Test-Revised, PANAS = Positive and Negative Affect Schedule.

**Table S2. Linear mixed-effects model predicting interoceptive accuracy with heart rate as a time-varying covariate**

| **Effect** | **df** |  | **F** | **p** |
| --- | --- | --- | --- | --- |
| Group | 1 |  | 0.13 | .720 |
| Time | 1 |  | 2.95 | .090 |
| Heart rate | 1 |  | 2.29 | .134 |
| Group × Time | 1 |  | 3.93 | .054 |

df = degrees of freedom; F = F value; p = p value.

**Table S3. Model comparison between the primary and heart-rate-adjusted mixed-effects models**

| **Model** | **Parameters** | **AIC** | **BIC** | **logLik** | **χ²** | **df** | **p** |
| --- | --- | --- | --- | --- | --- | --- | --- |
| Primary model: Group × Time | 6 | -73.24 | -58.80 | 42.62 |  |  |  |
| HR-adjusted model: Group × Time + HR | 7 | -73.64 | -56.80 | 43.82 | 2.40 | 1 | .121 |

df = degrees of freedom; χ² = chi-square statistic; p = p value; AIC = Akaike information criterion; BIC = Bayesian information criterion; logLik = log-likelihood; HR = heart rate.
